# Prevalence and Risk Factors Associated With Postpartum Depressive Symptoms Among Women in Vientiane Capital, Lao PDR

**DOI:** 10.3389/fpubh.2022.791385

**Published:** 2022-05-03

**Authors:** Amkha Xayyabouapha, Vanphanom Sychareun, Bui Thi Tu Quyen, Manivone Thikeo, Jo Durham

**Affiliations:** ^1^Faculty of Public Health, University of Health Sciences and Hanoi University of Public Health, Vientiane, Laos; ^2^Faculty of Public Health, University of Health Sciences, Vientiane, Laos; ^3^Biostatistics Department, Faculty of Fundamental Sciences, Hanoi University of Public Health, Vietnam, Laos; ^4^School of Public Health and Social Work, Queensland University of Technology, Brisbane, QLD, Australia

**Keywords:** prevalence, risk factors, postpartum depressive symptoms, Edinburgh Postnatal Depression Scale (EDPS), low-income country, Lao PDR

## Abstract

Postpartum depression (PPD), the onset of depressive episodes after childbirth, is the most common psychological condition following childbirth, and a global public health concern. If undiagnosed and/or untreated, postpartum depression can have negative effects on maternal and child health, however, there are few studies on the prevalence of postpartum depression in low- and middle-income countries. To contribute to filling this gap, this study examined the prevalence and risk factors associated with postpartum depressive symptoms among women after delivery in Vientiane Capital, Lao PDR. The study was a cross-sectional design, with multistage sampling used to identify women between 4 and 24 weeks after giving birth (*N* = 521). The Edinburgh Postnatal Depression Scale was used to identify women with postpartum depressive symptoms. Univariate and multivariate logistic regressions identified risk factors associated with postpartum depressive symptoms. The prevalence of postpartum depressive symptoms among participants was 21.3%. Associated factors were having at least 2–3 living children (AOR: 1.9, 95% CI: 1.1–3.0), experiencing mental health problems during pregnancy (AOR: 3.3, 95% CI: 1.4–7.6), experiencing conflicts with family members (AOR: 2.5, 95% CI: 1.5–4.0), the experience of intimate partner violence (AOR: 2.6, 95% CI: 1.3–5.5), and receiving moderate social support (AOR: 5.6, 95% CI: 3.2–10.0). In contexts where access to mental health specialists has severely constrained maternal and child healthcare providers at primary health care must be supported to develop the necessary skills to identify risk factors and symptoms and offer basic essential services for postpartum depressive symptom (PDS). The study identified a high proportion of mothers with postnatal depressive symptoms, highlighting the need to screen and treat mothers who present with PDS, as not doing so exposes mother and their children to a range of negative health and social outcomes. Addressing the stigma associated with mental health illness and mental health illness and domestic violence that prevents women from seeking healthcare, must also be developed, implemented, and evaluated.

## Introduction

Postpartum depression (PPD), the onset of depressive episodes after childbirth, is the most common psychological condition following childbirth, and a global public health concern (1, 2). A recent review of 565 studies from 80 different countries or regions, estimated the global prevalence of PPD to be 17.22% (95% CI 16.00–18.51) (1). This estimate is higher than the often-cited prevalence rate of 13% (95% CI: 12.3–13.4%), derived from a meta-analysis of studies from higher-income countries (3), and lower than the 19% prevalence rate for PPD obtained from studies of low- and middle-income countries (4). The review also found significant differences between geographic regions and confirmed low- and lower-middle-income countries carry the greatest PPD burden (1). Within Asia, the study found that Southern Asia had the highest prevalence (22.32, 95% CI 18.48–26.70), followed by Western Asia (19.83, 95% CI 17.33–22.58), Eastern Asia (17.39, 95% CI 16.09–18.77), and South-East Asia (13.53, 95% CI 11.00–16.52) (1).

Postpartum depressive symptoms (PDS) may occur at any time up to a year after childbirth, often peaking 4–6 weeks postpartum and disappearing spontaneously 2–6 months after giving birth but can last longer (5). Symptoms can include sadness, nausea, anxiety, irritability, decreased libido, feelings of isolation, and disturbances in appetite, energy, and sleep (1, 6). Thoughts of hurting oneself and/or the infant, are also common symptoms of PPD, which can have negative impacts on family relationships (7–9). Negative long-term consequences to the infant can include delayed cognitive, social, emotional, and physical development with potentially long-term effects (10–14). PDS, however, are often overlooked, resulting in late diagnosis and increased chances of aggravating PPD.

A variety of factors, including demographic (4, 15, 16), obstetric (17), and environmental factors (18) and it is a complex interplay of these factors that create vulnerability to PPD. Socio-demographic factors associated with PPD include younger age of mothers (4, 15), low education (17), unemployment (19), low income (17, 20, 21), and previous experiences of mental health disorders (20, 22, 23). Obstetric risk factors include complications during labor, or immediately postpartum, such as obstetric hemorrhage (24), unplanned pregnancies (24), and having two or more children (25). The evidence related to a number of children and PPD, however, is mixed, and it is unlikely to be an independent factor for developing PPD. Regarding the social environment, poor social support including emotional support and empathetic relations with family and community members (26) and experience of violence (27) is associated with an increased risk of PPD.

Cultural and family factors may be risk or protective factors for PDD. Family support including from mothers and mothers-in-law where the support provided meets the needs and expectations of the mother can be protective (28). Family and social support can however be a source of conflict, especially where there are differences in thinking and parenting concepts between older and mothers of more influential family members (28). In some countries, women are expected to go through a period of confinement postpartum, usually for a period of about 4 weeks during which there are often behavioral and dietary restrictions (29). In Southeast Asia, after giving birth mothers may rest for up to 1 month on a “hot bed” –a bed with a fire lit beneath it, which is thought to strengthen the health and accelerate the contraction of the uterus of a woman. During this time care is often provided by female relatives including mothers or mothers-in-law, often including the preparation of special foods and herbal tonics (29). This increased support temporarily changes the mother's role from caregiver to one who is cared for (30). The extent to which cultural practices in different countries are associated with PDD, however, is not well understood, with evidence on the role of confinement practices complicated by the heterogeneity of these practices (1, 30, 31). These cultural practices are also dynamic and influenced by factors such as changing economic systems, industrialization, globalization, and immigration (30).

Despite concern about PPD, an understanding of its prevalence in the Lao PDR, a lower-middle-income in South-East Asia, with few mental health services, is scant. A 2018 report on psychiatric services revealed there were 2,421 patients with a mental health condition, of which around 10% (240 cases) were diagnosed with depression, of whom 64% were women. The number of women with PPD specifically however was not reported (32). The reported number of cases is also likely to be significantly lower than the true number of people experiencing mental health disorders due to low awareness of mental health disorders, low health-seeking behaviors, stigma related to mental health disorders, and few mental health specialists or services in the country (32). There are, for example, only two hospitals in Lao PDR, both in the capital city of Vientiane, that have psychiatry departments, but neither of these departments has psychiatric specialists (32). The only available information on PDD comes from an unpublished study in 2017 conducted among women 6 weeks after giving birth in Odoumxay Province which indicated a prevalence of 15.2% PPD (21). To build on this work, this study was conducted to determine the prevalence and risk factors associated with PPD among women in Vientiane Capital, Lao PDR.

## Materials

This study was an analytical, cross-sectional, quantitative design, conducted among 48 communities in urban and peri-urban areas within Vientiane Capital. The Capital consists of nine districts, only three of which are entirely urban. A total of 77.9% of the population live in these urban districts, among whom are 22 births and 4.9 deaths per 1,000 population (33).

### Study Participants

The inclusion criteria for participants were mothers between 4- and 24-weeks postpartum and living in the selected communities at the time of the study. The sample size was calculated based on a single population formula, corresponding to a 95% confidence level at Z = 1.96, and an estimated prevalence of PDS from previous research of 15% (*p* = 0.15) (21). The absolute precision was d = 0.04, with 1.7 allowed for the design effect and 5% for the non-response rate, producing a sample size of 540 mothers. Multistage sampling was used to identify potential participants and maximize opportunities to recruit women from different socio-demographic backgrounds. One urban (Sisattanak) and one peri-urban district (Hadsayfong) within Vientiane Capital were selected using simple random sampling. Simple random sampling was then applied to select villages in each district, with 18 villages selected from the Sisattanak district and 30 from the Hadsayfong district. A total of 260 mothers from the Sisattanak district and 261 mothers from the Hadsayfong district were enrolled in the survey, with all interviews being completed within 1 month.

### Measurement of Variables

The independent variables consisted of socio-demographic characteristics, obstetric, pregnancy and pediatric health factors, and family relationship factors. Socio-demographic variables included maternal age, level of education, occupation, ethnicity, religion, marital status, and self-reported household financial status. The obstetric, pregnancy, and pediatric health variables included number of pregnancies, number of deliveries, number of abortions, number of living children, history of stillbirths, pregnancy planning, number of antenatal care visits, complications during pregnancy, complications during the postpartum period, mental health problems during pregnancy, mode of delivery, gestational age of birth, infant feeding, and newborn health status. Family relationship factors included living arrangements, conflicts with family members, social support, and intimate partner violence (IPV).

Perceived social support was measured by the Multidimensional Scale of Perceived Social Support (34). The scale consists of 12 questions, each with a 7-point Likert scale and questions related to family, friends, and significant other support. The overall average score for social support was determined as follows: <3 is low, 3–5 is moderate and > 5 is high social support (34). The Women Abuse Screening Tool (WAST) was used to assess IPV. The WAST includes 8 items, each scoring 1 for never or none, 2 for sometimes, and 3 for a lot or often. Total scores range from 08 to 24, with the recommended cut-off of 13 applied to indicate the presence of abuse (35).

The dependent variable was the occurrence of PPD derived using the Edinburgh Postpartum Depression Scale (EPDS). The EPDS is the most common measure used to screen for depression related to childbearing (1), it is not, however, a clinical diagnostic tool and should be complemented with clinical assessment. The EPDS consists of ten questions each with four choices, scoring 0, 1, 2, and 3 for questions 1, 2, and 4, and reverse scoring 3, 2, 1, and 0 for questions 3, 5–10. The total scores range from 0 to 30, with respondents scoring ≥ 10 considered as having depressive symptoms (36).

### Statistical Analysis

This study used EpiData to enter the data and Stata 14 for analysis. Descriptive statistics were applied to analyze the frequency and percentage of the independent and outcome variables. Tests for significance were undertaken using univariate analysis, and the results of these with a *p* < 0.2 were included in the multivariate logistic regression. A backward stepwise selection was applied with the level of significance for variables to remain in the final model set at *p* < 0.05, a 95% confidence interval for estimating the precision of the odds ratio, and the respective variables with significant associations.

### Ethical Considerations

Ethical approval was obtained from the Research Ethics Committee of the University of Health Sciences in Lao PDR (No 190/19), and the Ethical Review Board for Biomedical Research, Hanoi University of Public Health (No: 431/2019/YTCC-HD3). The research objectives, methodology, and potential risks were made known to each respondent and written consent was obtained before the interview. Participants were free to withdraw from the study at any time. To respect the confidentiality of participants, the names of respondents were not included in any results, and the data was securely maintained.

## Results

### Socio-Demographic Profile of Participants

A total of 521 postpartum mothers completed the survey (50.1% from the peri-urban district and (49.9% from the urban district. Just over half of the participants (53.7%) were aged 21 to 30 years, with a mean age of 27.5 (SD = 5.8). Most participants (97.7%) were married, and over half (58.8%) were unemployed at the time of the study (Table 1).

**Table 1:** Socio-demographic-economic profiles of the participants.

**Characteristics (*n*: 521)**	**Frequency**	**Percentage**
**District**		
Sisattanak (urban)	260	49.9
Hadsayfong (peri-urban)	261	50.1
**Maternal age (Mean 27.5 years, SD 5.8 years, Min: 16, Max: 42)**		
16–20	78	15
> 20–30	280	53.7
> 30–35	116	22.3
36–42	47	9
**Level of education**		
Never been in school/Primary school	128	24.6
Lower/ Upper secondary school	217	41.7
Vocational/Higher vocational certificate/Bachelor's	176	33.7
degree or higher		
**Occupation**		
Employed	215	41.2
Unemployed	306	58.8
**Ethnicity**		
Lao-Tai	443	85.2
Other ethnicity	78	14.8
**Religion**		
Buddhism	440	84.4
Other religion	81	15.6
**Marital status**		
Married	509	97.7
Other (single/divorced/separated/widow)	12	2.3
**Financial status**		
Enough with savings	198	38
Enough without savings	215	41.3
Not enough	108	20.7

### Obstetric, Pregnancy, and Pediatric Health Factors

Around half of the participants (51.4%) had two-three living children. The minimum number of children was one and a maximum of six. A total of 56.3% of the participating mothers were between 13 and 24 weeks after childbirth when interviewed. Over half (54.1%) had planned their last pregnancy, 6% reported mental health problems during their last pregnancy, and most (80.4%) reported a normal vaginal delivery. Almost all (94%) had healthy babies, 9% had a preterm birth (≤ 36 weeks), and 55% exclusively breastfed their babies.

### Mothers' Relationships With Family Members

Nearly 40% of mothers reported sometimes having conflicts with family members. Over half (56.1%), reported receiving moderate social support, and just under 9% had experienced IPV (Table 2).

**Table 2:** Family relationship characteristics of participants.

**Family relationship factors**	**Frequency**	**Percentage**
**Living arrangement**		
Living apart from family of birth/in-laws	219	42.0
Living with family of birth	202	38.8
Living with family-in-law	100	19.2
**Conflicts with family members**		
Never	295	56.6
Sometimes	208	39.9
Often	18	3.5
**Social support (Min: 3, Max: 6.83)**		
High support (6–max)	229	43.9
Moderate support (3–5)	292	56.1
Low support (min−2.9)	0	0
**Intimate partner violence WAST score (Min: 12, Max: 24)**		
Non-present IPV <13	477	91.5
Present IPV ≥ 13	44	8.5

### Prevalence of Postpartum Depressive Symptoms

Among the 521 mothers, 111 (21.3%) met the criteria of PDS as measured by an EPDS score of ≥10 (Table 3).

**Table 3:** Prevalence of postpartum depressive symptoms.

	**EPDS score** **<** **10**		**EPDS scores** **≥10**	
	**Frequency**	**Percentage**	**Frequency**	**Percentage**
Postpartum depressive symptoms in two districts	410	78.7	111	21.3
Sisattanak district	202	77.7	58	22.3
Hadsayfong district	208	79.7	53	20.3

### Risk Factors Associated With Postpartum Depressive Symptoms

The 18 independent variables that had a *p* < 0.2 in the univariate analysis—level of education, ethnicity, religion, financial status, planning of last pregnancy, number of antenatal care visits in recent pregnancy, complications during last pregnancy, mental health problems during last pregnancy, mode of delivery, number of pregnancies, number of deliveries, number of abortions, number of living children, gestational age of birth, conflict with family members, living arrangement, intimate partner violence, and social support—were entered into the multivariate analysis. The variables significantly associated with depressive symptoms in the multivariate logistic regression were mothers who had 2–3 living children (AOR: 1.9, 95% CI: 1.1–3.0), mothers who had mental health problems during pregnancy (AOR: 3.3, 95% CI: 1.4–7.6), mothers who experienced family conflicts (AOR: 2.5, 95% CI: 1.5–4.0), women who received moderate social support (AOR: 5.6, 95% CI: 3.2–10.0), and women who had experienced IPV (AOR: 2.6, 95% CI: 1.3–5.5) (Table 4).

**Table 4:** Risk factors associated with PDS among participants.

**Variables**	**EPDS <10**	**EPDS ≥10**	**COR**	***p*-value**	**AOR**	***P*-value**
	**F (%)**	**F (%)**	**(95% CI)**		**(95% CI)**	
**Number of living children**
1 child	203 (83.5)	40 (16.5)	1		1	
2–3 children	200 (74.6)	68 (25.4)	1.7 (1.1–2.6)	0.014	1.9 (1.1–3.0)	0.009*
4–6 children	7 (70)	3 (30)	2.2 (0.5–8.7)	0.2	2.6 (0.5–12.1)	0.2
**Mental health problems during pregnancy**
No	395 (80.9)	95 (19.4)	1		1	
Yes	15 (48.4)	16 (51.6)	4.4 (2.2–9.2)	0.000	3.3 (1.4–7.6)	0.005*
**Conflicts with family members**
Never	152 (85.2)	43 (28.8)	1		1	
Sometimes/Often	158 (69.9)	68 (30.1)	2.5 (1.6–3.8)	0.000	2.5 (1.5–4.0)	0.000*
**Social support**
High social support	212 (92.6)	17 (7.4)	1		1	
Moderate social support	198 (67.8)	94 (32.2)	5.9 (3.4–10.2)	0.000	5.6 (3.2–10)	0.000*
**IPV**
Non-present IPV	389 (81.5)	88 (18.5)			1	
Present IPV	21 (47.7)	23 (52.3)	4.8 (2.5–9.1)	0.000	2.6 (1.3–5.5)	0.007*

The symbol * indicates statistically significant values at p < 0.005.

## Discussion

This study investigated the prevalence of PDS and associated risk factors among women in Vientiane Capital, Lao PDR. The study revealed that around one-fifth of participants had depressive symptoms during the postpartum period as measured by the EPDS. This figure is higher than that found in Odoumxay in the north of Lao PDR (15.2%) (21) and findings from neighboring Thailand where the prevalence of PPD was reported to be 8.4% (37). These differences may be due to differences in study designs (e.g., sample size, parity, socio-economic class), and in outcome measurement (e.g., screening questionnaires used and the timing). The study in northern Lao PDR, for example was conducted among women from 1 to 6 weeks after delivery, whereas the sample in this study was postpartum mothers between 4 and 24 weeks. The study in Thailand used an EPDS score of ≥ 13 as an indicator of having depressive symptoms, whereas in the present study we applied a score ≥ 10 (36). The prevalence of PPS in the present study is however comparable to a study in rural Bangladesh (22%) (38), studies in South Asia (39–41), although higher the those reported for South-East Asia reported in Wang et al. (1) global review (13.53, 95% CI 11.00–16.52) and in higher-income countries. Consistent with studies in higher-income countries and within Asia, postnatal depression was associated with antenatal experiences of poor mental health, family and marital disharmony, exposure to IPV, and not enough social support from partners, families, and friends (39–43). Having 2–3 living children was also associated with PDS which may be due to women with more children having many activities and financial concerns. This finding is contradicted in other research, however, including China (44) and Vietnam (45), and number of children may not be an independent factor for developing PDS and warrants further research.

This study indicates mothers who experience mental health problems during pregnancy are more likely to experience PDS. Similar findings have been reported in studies in Vietnam (46), Thailand (47), and South Korea (48). This finding may be due to hormonal imbalance during pregnancy and the postpartum period contributing to a relapse of depression (27). While results are mixed, an increasing body of research suggests PPD with onset proximal to childbirth, is a discrete depressive disorder, highlighting the importance of integrating screening, and mental health support into routine maternal care (49, 50). Integrating mental health into perinatal care requires providers are empowered with the skills necessary for them to offer basic but essential services for PDS, especially in lower- and middle-income countries where there is often very limited access to mental health specialists. Research indicates non-physician primary care providers and midwives, can deliver basic evidence-based interventions for depression occurring in perinatal women in non-specialist settings, as described in the mental health Gap Action Programme Intervention Guide (46–48, 50).

Affirming other studies, lack of social support and experience of frequent conflicts with family members was a risk factors for PDD (26, 51–53). It is possible conflicts occur in societies such as Lao PDR where patriarchal cultural norms are predominant and mothers-in-law may have a significant influence on how the newborn and mother should be taken care of. In countries amid a demographic transition, such as Lao PPDR, there may also be conflicts related to more traditional and more contemporary infant care, such as the feeding of colostrum to the newborn (54). Patriarchal cultural norms may also contribute to IPV, and as elsewhere, IPV was associated with PDS (24, 55) and is likely to be explained by the social, emotional, and physical isolation often felt by women exposed to IPV and the unpredictability of the abuser (55–57). This underlines the need for healthcare providers to include discussion of social support and family context in their consults with perinatal women. Low public awareness and stigma about mental disorders, and availability and acceptability of health services are also salient and symptoms of PDS may go unrecognized or be attributed to non-medical causes (49).

This study has some limitations. The study was conducted in only two districts, one urban and one peri-urban, within Vientiane Capital and cannot be considered representative of all districts in Vientiane Capital. As a cross-sectional study, we could only examine factors associated with PDS and cannot make claims about causality. Further, the EPDS provides an indicator of symptoms and is not a clinical diagnosis of depression, nor has the Lao version of the EPDS version been validated in Lao PDR, although in this study good internal consistency was demonstrated (Cronbach's alpha 0.75). We also recognize the limitation of self-report measures which may be prone to social desirability bias, especially given the stigma associated with mental illness. We also cannot exclude the possibility of recall bias. Also of note, this study was conducted before the onset of the COVID-19 pandemic, and it is possible that since then rates of PDS are higher (58, 59), underscoring the need for early detection and support for those at risk of, or experiencing, PPD.

## Conclusion

The study identified a high proportion of mothers with postnatal depressive symptoms in a context where there is limited support for mental health disorders. This highlights the need to screen and treat mothers who present with PDS, as not doing so exposes mother and their children to a range of negative health and social outcomes. In contexts where access to mental health specialists has severely constrained maternal and child healthcare providers at primary health care must be supported to develop the necessary skills to identify risk factors and symptoms and offer basic essential services for PDS. Addressing the stigma associated with mental health illness and domestic violence that prevents women from seeking healthcare, must also be developed, implemented, and evaluated.

## Data Availability Statement

The raw data supporting the conclusions of this article will be made available by the authors, without undue reservation.

## Ethics Statement

The studies involving human participants were reviewed and approved by the Research Ethic Committee of the University of Health Sciences, Lao PDR. Written informed consent for participation was not required for this study in accordance with the national legislation and the institutional requirements.

## Author Contributions

AX developed the research proposal, designed the instrument, collected data in the field sites analyzed the data, and wrote the draft manuscript. VS, MT, BQ, and JD contributed to the statistical analysis, interpretation of results, and commented and made revisions to previous versions of the manuscript. All authors read and approved the final manuscript.

## Funding

This study was funded by the Ministry of Health of the Lao PDR and the LEARN Project.

## Conflict of Interest

The authors declare that the research was conducted in the absence of any commercial or financial relationships that could be construed as a potential conflict of interest.

## Publisher's Note

All claims expressed in this article are solely those of the authors and do not necessarily represent those of their affiliated organizations, or those of the publisher, the editors and the reviewers. Any product that may be evaluated in this article, or claim that may be made by its manufacturer, is not guaranteed or endorsed by the publisher.

## References

[1] Wang Z Liu J Shuai H Cai Z Fu X Liu Y. Mapping global prevalence of depression among postpartum women. Transl Psychiatry. (2021) 11:543. 10.1038/s41398-021-01663-6 34671011 PMC8528847

[2] WHO. Women and Health: Today's Evidence Tomorrow's Agenda. Geneva: World Health Organization (2009).

[3] O'Hara MW Swain AM. Rates and risk of postpartum depression-a meta-analysis. Int Rev Psychiatry. (1996) 8:37–54. 10.3109/09540269609037816

[4] Fisher J Mello MCd Patel V Rahman A Tran T Holton S. Prevalence and determinants of common perinatal mental disorders in women in low- and lower-middle-income countries: a systematic review. Bull World Health Organ. (2012) 90:139–49. 10.2471/BLT.11.091850 22423165 PMC3302553

[5] Lee TSD Chung KHT. Postnatal depression: an update. Best Pract Res Clin Obstet Gynaecol. (2007) 21:183–91. 10.1016/j.bpobgyn.2006.10.003 17157072

[6] Robertson E Grace S Wallington T Stewart DE. Antenatal risk factors for postpartum depression: a synthesis of recent literature. Gen Hosp Psychiatry. (2004) 26:289–95. 10.1016/j.genhosppsych.2004.02.006 15234824

[7] Howard LM Flach C Mehay A Sharp D Tylee A. The prevalence of suicidal ideation identified by the edinburgh postnatal depression scale in postpartum women in primary care: findings from the RESPOND trial. BMC Pregnancy Childbirth. (2011) 11:57. 10.1186/1471-2393-11-57 21812968 PMC3161003

[8] Orsolini L Valchera A Vecchiotti R Tomasetti C Iasevoli F Fornaro M. Suicide during perinatal period: epidemiology, risk factors, and clinical correlates. Front Psychiatry. (2016) 7:138. 10.3389/fpsyt.2016.00138 27570512 PMC4981602

[9] Wisner KL Sit DK McShea MC Rizzo DM Zoretich RA Hughes CL. Onset timing, thoughts of self-harm, and diagnoses in postpartum women with screen-positive depression findings. JAMA Psychiatry. (2013) 70:490–8. 10.1001/jamapsychiatry.2013.87 23487258 PMC4440326

[10] Madeghe BA Kimani VN Vander Stoep A Nicodimos S Kumar M. Postpartum depression and infant feeding practices in a low income urban settlement in Nairobi-Kenya. BMC Res Notes. (2016) 9:506. 10.1186/s13104-016-2307-9 27931248 PMC5146885

[11] Patel V DeSouza N Rodrigues M. Postnatal depression and infant growth and development in low income countries: a cohort study from Goa, India. Arch Dis Child. (2003) 88:34–7. 10.1136/adc.88.1.34 12495957 PMC1719257

[12] Paulson JF Dauber S Leiferman JA. Individual and combined effects of postpartum depression in mothers and fathers on parenting behavior. Pediatrics. (2006) 118:659–68. 10.1542/peds.2005-2948 16882821

[13] Surkan PJ Kawachi I Ryan LM Berkman LF Carvalho Vieira LM Peterson KE. Maternal depressive symptoms, parenting self-efficacy, and child growth. Am J Public Health. (2008) 98:125–32. 10.2105/AJPH.2006.108332 18048782 PMC2156066

[14] Surkan PJ Kennedy CE Hurley KM Black MM. Maternal depression and early childhood growth in developing countries: systematic review and meta-analysis. Bull World Health Organ. (2011) 89:607–15. 10.2471/BLT.11.088187 21836759 PMC3150769

[15] Kheirabadi G-R Maracy M-R Barekatain M Casey PR. Risk factors of postpartum depression in rural areas of Isfahan Province, Iran. Arch Iran Med. (2009) 12:461–7. 19722767

[16] Hidaka BH. Depression as a disease of modernity: explanations for increasing prevalence. J Affect Disord. (2012) 140:205–14. 10.1016/j.jad.2011.12.036 22244375 PMC3330161

[17] Ghaedrahmati M Kazemi A Kheirabadi G Ebrahimi A Bahrami M. Postpartum depression risk factors: a narrative review. J Educ Health Promot. (2017) 6:60. 10.4103/jehp.jehp_9_16 28852652 PMC5561681

[18] O'Hare C O'Sullivan V Flood S Kenny RA. Seasonal and meteorological associations with depressive symptoms in older adults: geo-epidemiological study. J Affect Disord. (2016) 191:172–9. 10.1016/j.jad.2015.11.029 26655862

[19] Sampson M Duron JF Mauldin RL Kao D Davidson M. Postpartum depression, risk factors, and child's home environment among mothers in a home visiting program. J Child Fam Stud. (2017) 26:2772–81. 10.1007/s10826-017-0783-8

[20] Ghosh A Goswami S. Evaluation of post partum depression in a tertiary hospital. The J Obstet Gynecol India. (2011) 61:528–30. 10.1007/s13224-011-0077-9 23024522 PMC3257346

[21] Inphouvieng C. Prevalence of postpartum depression among women in Oudomxai Province (Master of Public Health Thesis). University of Health Sciences, Vientiane Capital, Lao PDR (2017).

[22] Chojenta CL Lucke JC Forder PM Loxton DJ. Maternal health factors as risks for postnatal depression: a prospective longitudinal study. PLoS ONE. (2016) 11:e147246. 10.1371/journal.pone.0147246 26785131 PMC4718697

[23] Davey HL Tough SC Adair CE Benzies K. Risk factors for sub-clinical and major postpartum depression among a community cohort of Canadian women. Matern Child Health J. (2011) 15:866–75. 10.1007/s10995-008-0314-8 18256913

[24] Ahmad NA Silim UA Rosman A Mohamed M Chan YY Kasim NM. Postnatal depression and intimate partner violence: a nationwide clinic-based cross-sectional study in Malaysia. BMJ Open. (2018) 8:e20649. 10.1136/bmjopen-2017-020649 29764882 PMC5961592

[25] Desai N Mehta R Ganjiwale J. Study of prevalence and risk factors of postpartum depression. Natl J Med Res. (2012) 2:194–8.

[26] Roomruangwong C Epperson CN. Perinatal depression in Asian women: prevalence, associated factors, and cultural aspects. Asian Biomed. (2011) 5:179–93. 10.5372/1905-7415.0502.024

[27] Adamu AF Adinew YM. Domestic violence as a risk factor for postpartum depression among Ethiopian women: facility based study. Clin Pract Epidemiol Ment Health. (2018) 14:109–19. 10.2174/1745017901814010109 29997678 PMC5971200

[28] Callister LC Beckstrand RL Corbett C. Postpartum depression and culture: pesado Corazon. Am J Matern Child Nurs. (2010) 35:254–61. 10.1097/NMC.0b013e3181e597bf 20551841

[29] de Boer HJ Lamxay V Björk L. Steam sauna and mother roasting in Lao PDR: practices and chemical constituents of essential oils of plant species used in postpartum recovery. BMC Complement Altern Med. (2011) 11:128. 10.1186/1472-6882-11-128 22171719 PMC3265423

[30] Dennis C-L Fung K Grigoriadis S Robinson GE Romans S Ross L. Traditional postpartum practices and rituals: a qualitative systematic review. Womens Health. (2007) 3:487–502. 10.2217/17455057.3.4.487 19804024

[31] Bina R. The impact of cultural factors upon postpartum depression: a literature review. Health Care Women Int. (2008) 29:568–92. 10.1080/07399330802089149 18569045

[32] Ministry of Health. Report on Psychiatric Service Deliveries in 2018. Vientiane: Ministry of Health (2018).

[33] Lao Statistics Bureau,. Results of Population Housing Census 2015. (2015). Available online at: https://www.lsb.gov.la/pdf/PHC-ENG-FNAL-WEB.pdf.

[34] Zimet GD Dahlem NW Zimet SG Farley GK. The multidimensional scale of perceived social support. J Pers Assess. (1988) 52:30–41. 10.1207/s15327752jpa5201_2 2280326

[35] Brown JB Lent B Schmidt G Sas G. Application of the woman abuse screening tool (WAST) and WAST-short in the family practice setting. J Family Pract. (2000) 49:896–903. 10.1037/t04577-000 11052161

[36] Cox JL Holden JM Sagovsky R. Detection of postnatal depression: development of the 10-item edinburgh postnatal depression scale. Br J Psychiatry. (1987) 150:782–6. 10.1192/bjp.150.6.782 3651732

[37] Panyayong B. Postpartum depression among Thai women: a national survey. J Med Assoc Thai. (2013) 96:761–7. 24319843

[38] Gausia K Fisher C Ali M Oosthuizen J. Magnitude and contributory factors of postnatal depression: a community-based cohort study from a rural subdistrict of Bangladesh. Psychol Med. (2009) 39:999–1007. 10.1017/S0033291708004455 18812008

[39] Patel V Rodrigues M Gender DeSouza N. Poverty and post-natal depression: a cohort study from Goa, India. Am J Psychiatry. (2002) 159:43–7. 10.1176/appi.ajp.159.1.43 11772688

[40] Rahman A Iqbal Z Harrington R. Life events, social support and depression in childbirth: perspectives from a rural community in the developing world. J Arch Gen psychiatry. (2003) 33:1161. 10.1017/S0033291703008286 14580070

[41] Regmi S Sligl W Carter D Grut W Seear M. A controlled study of postpartum depression among Nepalese women: validation of the Edinburgh postpartum depression scale in Kathmandu. J Trop Med Int Health. (2002) 7:378–82. 10.1046/j.1365-3156.2002.00866.x 11952955

[42] Gomez-Beloz A Williams MA Sanchez SE Lam N. Intimate partner violence and risk for depression among postpartum women in Lima, Peru. J Violence. (2009) 24:380–98. 10.1891/0886-6708.24.3.380 19634363

[43] Sawyer A Ayers S Smith H. Pre-and postnatal psychological wellbeing in Africa: a systematic review. J Affect Disord. (2010) 123:17–29. 10.1016/j.jad.2009.06.027 19635636

[44] Xiong R Deng A Wan B Liu Y. Prevalence and factors associated with postpartum depression in women from single-child families. Int J Gynecol Obstetric. (2018) 141:194–9. 10.1002/ijgo.12461 29412451

[45] Do TKL Nguyen TTH Pham TTH. Postpartum depression and risk factors among Vietnamese women. Biomed Res Int. (2018) 2018:4028913. 10.1155/2018/4028913 30320133 PMC6167583

[46] Tho Tran N Nguyen HTT Nguyen HD Ngo TV Gammeltoft T Rasch V. Emotional violence exerted by intimate partners and postnatal depressive symptoms among women in Vietnam: a prospective cohort study. PLoS ONE. (2018) 13:e0207108. 10.1371/journal.pone.0207108 30412609 PMC6226195

[47] Petpornprapas E Lotrakul M. Postpartum depression: its relationship to childbirth and child health at Ramathibodi Hospital. J Psychiatr Assoc Thailand. (2009) 54:29–36. Availalble online at: https://he01.tci-thaijo.org/index.php/JPAT/article/view/7523

[48] Park J-h Karmaus W Zhang H. Prevalence of and risk factors for depressive symptoms in Korean women throughout pregnancy and in postpartum period. Asian Nurs Res. (2015) 9:219–25. 10.1016/j.anr.2015.03.004 26412626

[49] Batt MM Duffy KA Novick AM Metcalf CA Epperson CNE. Is postpartum depression different from depression occurring outside of the perinatal period? A review of the evidence. Focus. (2020) 18:106–19. 10.1176/appi.focus.20190045 33162848 PMC7587887

[50] Gelaye B Rondon MB Araya R Williams MA. Epidemiology of maternal depression, risk factors, and child outcomes in low-income and middle-income countries. J Lancet Psychiatry. (2016) 3:973–82. 10.1016/S2215-0366(16)30284-X 27650773 PMC5155709

[51] Murray L Dunne MP Van Vo T Anh PN Khawaja NG Cao TN. Postnatal depressive symptoms amongst women in Central Vietnam: a cross-sectional study investigating prevalence and associations with social, cultural and infant factors. BMC Pregnancy Childbirth. (2015) 15:234. 10.1186/s12884-015-0662-5 26423807 PMC4590693

[52] Stapleton LRT Schetter CD Westling E Rini C Glynn LM Hobel CJ. Perceived partner support in pregnancy predicts lower maternal and infant distress. J Fam Psychol. (2012) 26:453. 10.1037/a0028332 22662772 PMC3992993

[53] Elsenbruch S Benson S Rücke M Rose M Dudenhausen J Pincus-Knackstedt MK. Social support during pregnancy: effects on maternal depressive symptoms, smoking and pregnancy outcome. J Human Reproduct. (2007) 22:869–77. 10.1093/humrep/del432 17110400

[54] Lee HM Durham J Booth J Sychareun V. A qualitative study on the breastfeeding experiences of first-time mothers in Vientiane, Lao PDR. BMC Pregnancy Childbirth. (2013) 13:223. 10.1186/1471-2393-13-223 24304510 PMC4235169

[55] Campbell JC Lewandowski LA. Mental and physical health effects of intimate partner violence on women and children. J Psychiatr Clin North America. (1997) 20:353–74. 10.1016/S0193-953X(05)70317-8 9196919

[56] Ludermir AB Lewis G Valongueiro SA de Araújo TVB Araya R. Violence against women by their intimate partner during pregnancy and postnatal depression: a prospective cohort study. J Lancet. (2010) 376:903–10. 10.1016/S0140-6736(10)60887-2 20822809

[57] Zhang Y Zou S Cao Y Zhang Y. Relationship between domestic violence and postnatal depression among pregnant Chinese women. Int J Gynecol Obstet. (2012) 116:26–30. 10.1016/j.ijgo.2011.08.011 22024214

[58] Zanardo V Manghina V Giliberti L Vettore M Severino L Straface G. Psychological impact of COVID-19 quarantine measures in northeastern Italy on mothers in the immediate postpartum period. Int J Gynecol Obstet. (2020) 150:184–8. 10.1002/ijgo.13249 32474910 PMC9087548

[59] Mariño-Narvaez C Puertas-Gonzalez JA Romero-Gonzalez B Peralta-Ramirez MI. Giving birth during the COVID-19 pandemic: the impact on birth satisfaction and postpartum depression. Int J Gynecol Obstet. (2021) 153:83–8. 10.1002/ijgo.13565 33368216 PMC9087776

